# Splenic inflammatory pseudotumor-like follicular dendritic cell sarcoma: a case report with imaging features and literature review

**DOI:** 10.3389/fonc.2026.1686209

**Published:** 2026-02-11

**Authors:** Ruimiao Shang, Shuxu Zhu, Xiaoying Tao

**Affiliations:** Department of Ultrasound, Affiliated Jinhua Hospital, Zhejiang University School of Medicine, Jinhua, Zhejiang, China

**Keywords:** case report, follicular dendritic cell sarcoma, image features, inflammatory pseudotumor, spleen

## Abstract

**Background:**

Follicular dendritic cell sarcoma (FDCS) is an extremely rare malignant neoplasm originating from immune follicular dendritic cells. Its inflammatory pseudotumor-like subtype (IPT-like FDCS) is even more uncommon due to morphological overlap with inflammatory pseudotumors. We report a case of splenic IPT-like FDCS and perform a systematic literature review to clarify the multimodal imaging characteristics of this rare entity.

**Case presentation:**

A 50-year-old asymptomatic man had an incidentally detected splenic mass during routine health screening. Ultrasound showed a well-defined hypoechoic splenic lesion. Magnetic resonance imaging revealed slightly hyperintense signals on both T1- and T2-weighted images, with progressive inhomogeneous enhancement and a peripheral hypointense ring on T2-weighted sequences. The patient underwent splenectomy, and IPT-like FDCS was confirmed via histopathology and immunohistochemistry.

**Conclusions:**

Splenic IPT-like FDCS typically presents with nonspecific or subclinical manifestations, making noninvasive imaging modalities crucial for diagnostic hint. Progressive heterogeneous enhancement and a peripheral hypointense ring on T2-weighted imaging may be valuable imaging features for identifying this rare malignancy.

## Introduction

Inflammatory pseudotumor-like follicular dendritic cell sarcoma (IPT-like FDCS) usually occurs in the liver and spleen, and to a lesser extent in the gastrointestinal tract, pancreas, and other organs (1, 2). IPT-like FDCS is more common in women, with an average age of 56.5 years (3). Because of its atypical imaging presentation, IPT-like FDCS is easily misdiagnosed as hemangioma, inflammatory pseudotumor, or other conditions. IPT-like FDCS was found for the first time in our hospital, and this article describes the discovery, diagnosis, and treatment of this disease in the context of the literature.

## Case presentation

A 50-year-old male patient presented to our hospital in October 2023 due to an incidentally detected splenic lesion during a routine physical examination. The patient denied any subjective symptoms and had no relevant medical history; physical examination yielded unremarkable findings.

**Figure 1:**
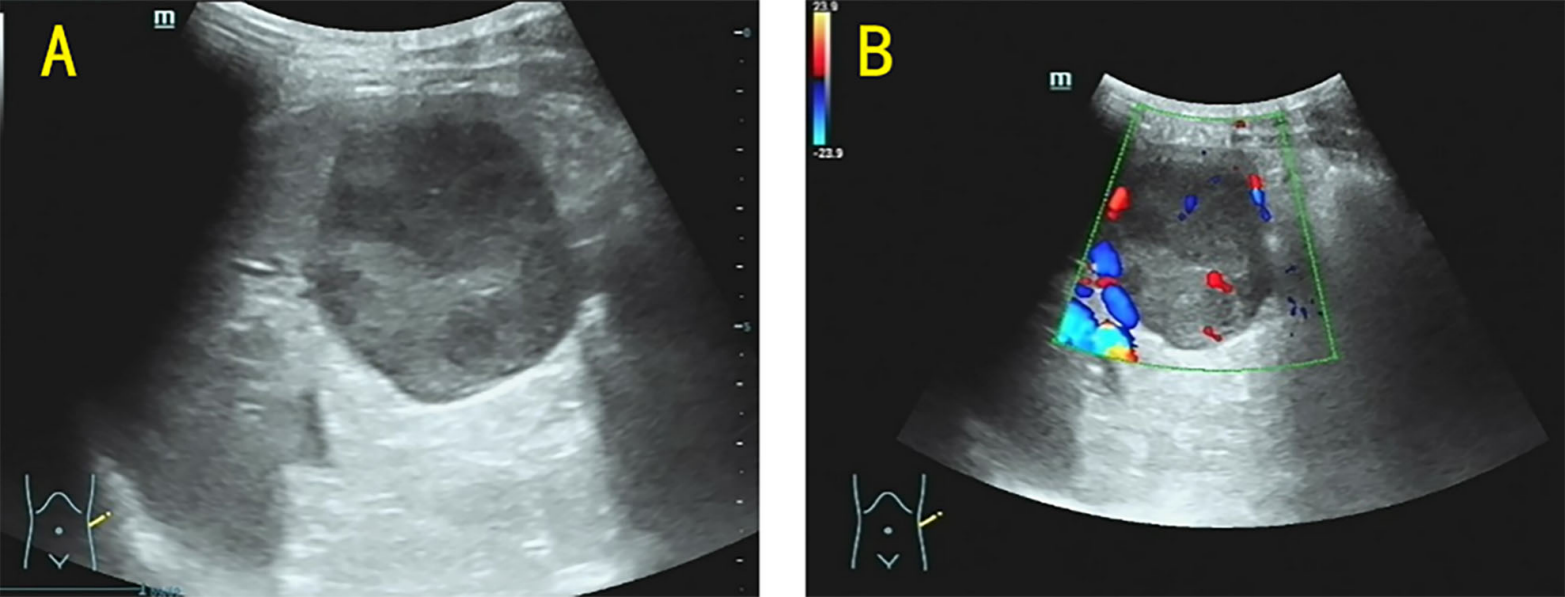
**(A)** Ultrasound revealed a hypoechoic mass of approximately 48 × 48 mm in size in the lower pole of the spleen, with well-defined borders. **(B)** The color Doppler revealed that blood flow signals were visible within the mass.

**Figure 2:**
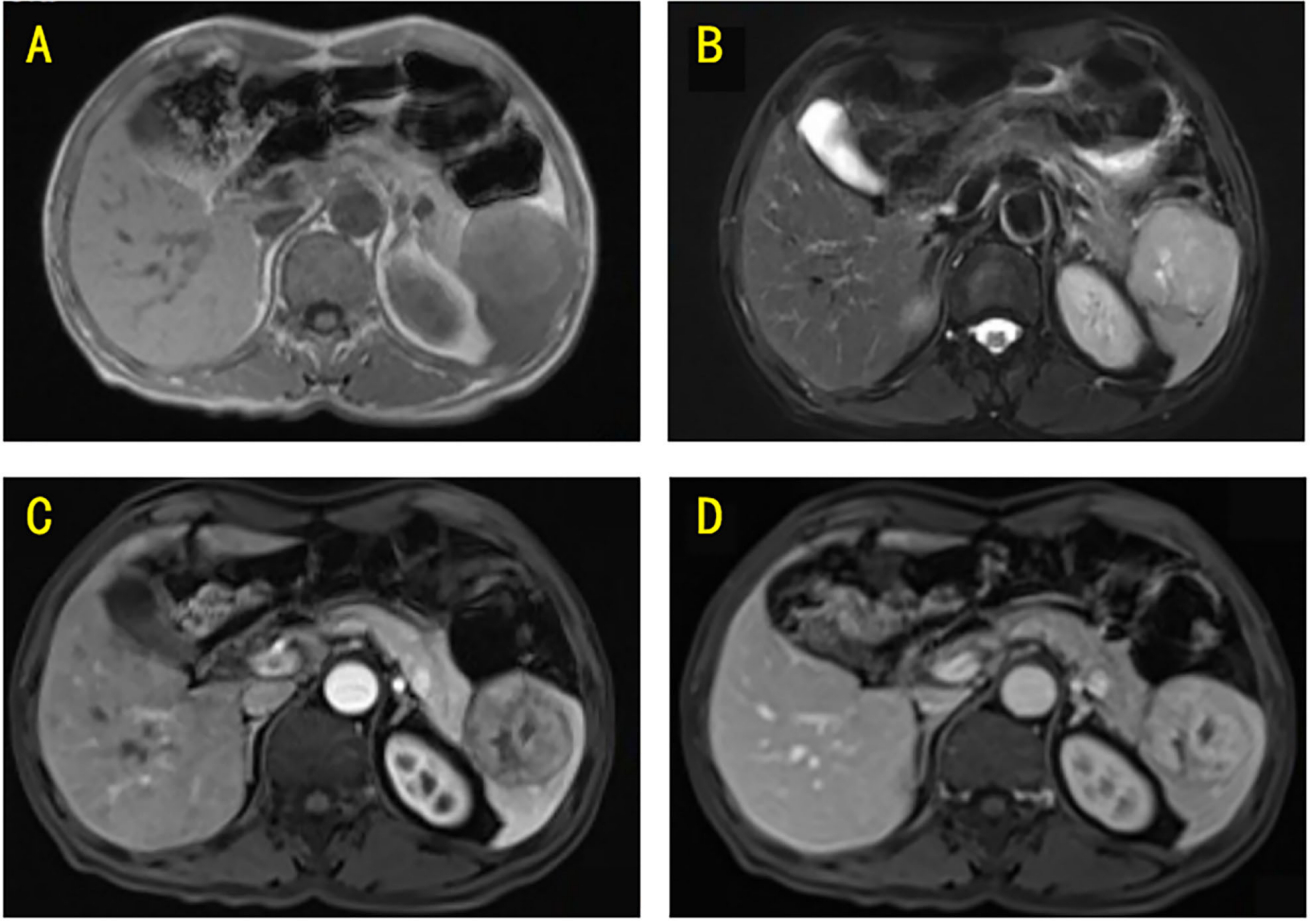
**(A)** T1-weighted MRI revealed a mass located within the spleen, which demonstrated a slightly hyperintense signal. **(B)** T2-weighted MRI showed that the mass presented a slightly hyperintense signal. **(C)** Post-contrast MRI (arterial phase) revealed homogeneous enhancement of the mass. **(D)** Post-contrast MRI (portal venous phase) showed further enhancement of the mass.

On the same day, ultrasonography was performed using a Mindray Resona R9TB system with an SC6-1U transducer, which revealed a mass measuring approximately 48 × 48 mm at the splenic inferior pole, with well-demarcated borders; color Doppler ultrasonography demonstrated visible intralesional blood flow signals (**Figure 1**). On the third day of hospitalization, magnetic resonance imaging (MRI) was performed on a Siemens Magnetom Avanto 1.5 T scanner, which revealed a splenic mass with slightly hyperintense signals on both T1-weighted and T2-weighted images, measuring approximately 57 × 54 mm. The lesion exhibited progressive heterogeneous enhancement after contrast administration (**Figure 2**).

**Figure 3:**
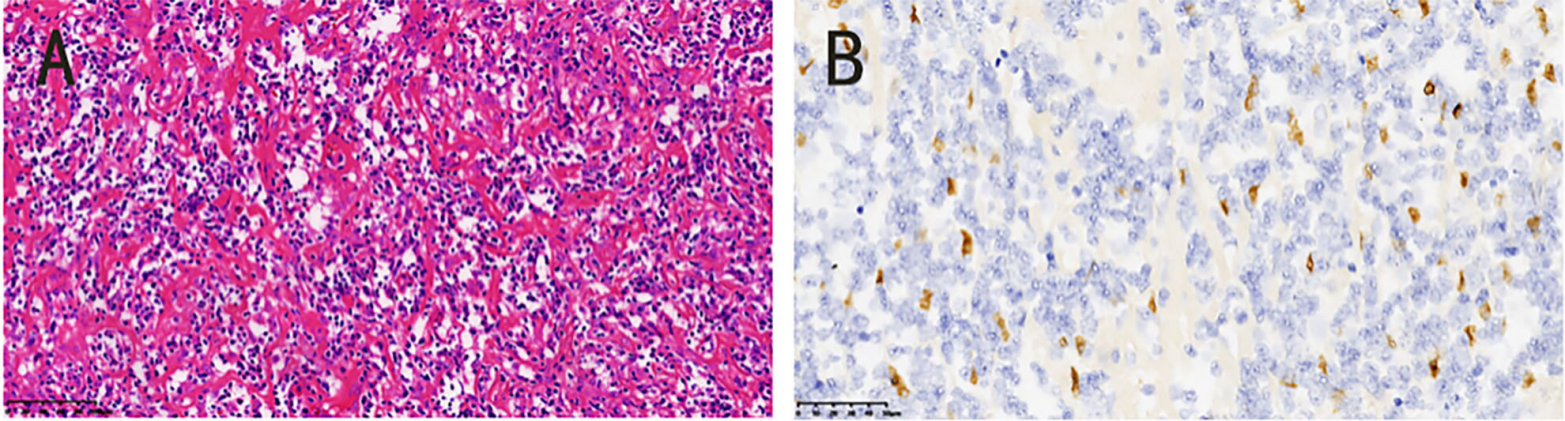
**(A)** The tumor cells were spindle-shaped and scattered in the background of chronic inflammation (HE 20×). **(B)** The nuclear chromatin was slightly vacuolar and some nucleoli could be seen: EBER(+), (HE 20×).

On the fourth day of hospitalization, laparoscopic splenectomy was successfully performed. Intraoperatively, a round, solid tumor was identified at the splenic inferior pole; sectioning of the tumor revealed a fish-flesh-like cut surface. The patient had an uneventful postoperative recovery and was discharged smoothly. The final diagnosis of IPT-like FDCS was established based on integrated histopathological and immunohistochemical findings. Immunohistochemical staining was conducted via the EnVision method on an automated immunohistochemistry analyzer (Leica Bond-MAX), with primary antibodies sourced from Beijing Zhongshan Golden Bridge Biotechnology Co., Ltd. and Fuzhou Maixin Biotechnology Co., Ltd. Immunohistopathological results showed the following immunoreactivity profile: CD21(+), CD23(+), CD35(+), Bcl-2(−), Bcl-6(−), CD10(−), CD68(+), CD138(+), CD3(−), CD30(−), CD43(+), CD38(+), CD34(−), and Ki-67 (20% positive). *In situ* hybridization confirmed Epstein–Barr virus-encoded small RNA (EBER) positivity (**Figure 3**).

At the 1-year follow-up, the patient was contacted via telephone. He reported no physical discomfort but did not return to the hospital for further imaging or laboratory follow-up examinations.

## Discussion

Follicular dendritic cell sarcoma (FDCS) is a rare mesenchymal neoplasm originating from follicular dendritic cells of the lymphoid tissue. Initially described in 1986 (4), this entity gained further recognition in 1996 when a morphologically distinct variant resembling inflammatory pseudotumor was reported, with proposed etiological links to Epstein–Barr virus (EBV) infection (5). The term “inflammatory pseudotumor-like follicular dendritic cell sarcoma” was subsequently formalized by Cheuk et al. in 2001 to characterize this clinicopathological subtype (6). Clinically, IPT-like FDCS typically follows an indolent course, and most patients are asymptomatic at initial presentation. Nonspecific symptoms such as localized pain or abdominal distension are observed in a minority of cases, often correlating with tumor size or anatomical involvement (7).

Several studies have demonstrated an association between inflammatory IPT-like FDCS and EBV, with latent membrane protein 1 detected in the majority of IPT-like FDCS cases (8). EBER *in situ* hybridization holds significant value in the definitive diagnosis of this condition (9). To systematically clarify the core characteristics of such cases and lay a foundation for subsequent analysis of diagnostic pitfalls, and management implications, we summarized and compared the key clinical and pathological data of reported cases in **Table 1**, focusing on multi-modality imaging findings [ultrasound, computed tomography (CT), and MRI], EBER status, and management strategies.

**Table 1:** Summary of clinical, pathological, multi-modality imaging findings, EBER status, and management strategies of reported splenic IPT-FDCS cases.

Case no.	Age	Presentation	Maximum diameter (mm)	Ultrasound findings	CT findings	MRI findings	EBER status	Management plan	Reference
1	67	Bleeding from the mouth, nose, and skin	60	NA	Patchy low-density shadow, moderate peripheral enhancement in the arterial phase, slightly reduced enhancement in the venous phase	NA	Positive	Laparoscopic total splenectomy	(9)
2	45	Soreness of the waist	29	Well-defined, homogeneous hypoechoic, no blood flow signal	Unclear boundary, isodensity, no mass was found in plain, moderate homogeneous enhancement	Well-defined, T1WI and T2WI short signal, no enhancement	NA	NA	(10)
3	40	No clinical symptoms	73	Well-defined, peripheral hypoechoic and dendritic hyperechoic centers, with a little blood flow signal	Unclear boundary, irregular low density, in the center and equal density in the periphery	Well-defined, T1WI and T2WI short signal, progressive enhancement with no enhancement in the center	NA	NA	(10)
4	81	No clinical symptoms	81	Well-defined, peripheral hypoechoic and dendritic hyperechoic centers, with a little blood flow signal	Unclear boundary, irregular low density, in the center and equal density in the periphery	NA	NA	NA	(10)
5	59	Dull pain in the left upper abdomen	150	NA	Well-defined, most cystic lesions and slight calcification in low density lesions, only moderate enhancement in peripheral and septal areas	Well-defined, T1WI and T2WI short signal, only moderate enhancement in peripheral and septal areas	NA	NA	(10)
6	54	Dull pain in the left upper abdomen	36	Well-defined, homogeneous hypoechoic, no blood flow signal	Unclear boundary, isodensity, no mass was found in plain, moderate homogeneous enhancement	NA	NA	NA	(10)
7	71	No clinical symptoms	45	Well-defined, hypoechoic peripheral and irregular hyperechoic central echoes, with a little blood flow signal	NA	Well-defined, T1WI and T2WI short signal, progressive enhancement with no enhancement in the center	NA	NA	(10)
8	67	No clinical symptoms	60	Well-defined, hypoechoic peripheral and irregular hyperechoic central echoes, with a little blood flow signal	Unclear boundary, irregular low density, in the center and equal density in the periphery, uneven moderate enhancement	NA	NA	NA	(10)
9	29	Dull pain in the left upper abdomen	110	Unclear boundary, a fluid hypoechoic region within the mass, as well as dotted and streaked blood hyposignals in and around the mass	Heterogeneous density with patchy, slightly hyperdense and poorly defined borders, the parenchymal portion of the tumor had progressive enhancement	Well-defined, mixed-signal, the tumor margin had an envelope-like structure and the parenchyma exhibited progressive enhancement	Positive	Open splenectomy	(11)
10	57	No clinical symptoms	50	Inhomogeneous hypoechoic mass with calcification	Circular isodensity with punctate calcifications, progressive enhancement	Well-defined, T2WI showed most of the lesions were slightly low-intensity shadows, T1WI showed most of the lesions were iso-intensity shadows, obvious enhancement in arterial and venous phases	Positive	Splenectomy	(12)
11	61	No clinical symptoms	100	Well-defined	Well-defined, isodense, subtle inhomogeneous enhancement in the early-phase images	Well-defined, T1WI isointense, T2WI mildly increased signal intensity, inhomogeneous enhancement	Positive	Open splenectomy	(13)
12	63	Rapid weight loss	124	NA	Inhomogeneous density and peripheral enhancement	NA	Positive	Splenectomy	(14)
13	59	Occasional left low back pain	45	NA	Low-density, mild, and sustained enhancement in each phase	NA	Positive	Partial splenectomy	(15)
14	64	Upper abdominal pain	72	NA	Hypodense mass with slight enhancement	NA	Positive	Laparoscopic splenectomy	(16)
15	61	No clinical symptoms	62	NA	Hypodense mass with slight enhancement	NA	Positive	Laparoscopic splenectomy	(16)
16	42	Left-sided flank pain	40	NA	Hypodense mass with slight enhancement	NA	Positive	Laparoscopic splenectomy	(16)
17	57	Upper abdominal pain	133	NA	Hypodense mass with slight enhancement	NA	Positive	Laparoscopic splenectomy	(16)
18	52	Back pain	37	NA	Hypodense mass with slight enhancement	NA	Positive	Laparoscopic splenectomy	(16)
19	58	No clinical symptoms	50	Well-marginated, round, hypoechoic	Well-defined, round, well-defined, progressive enhancement	NA	Positive	Splenectomy	(17)
20	70s	Acute abdominal pain	99	NA	Heterogeneous, central attenuation	NA	Positive	Splenectomy	(18)
21	72	Left epigastralgia	70	NA	Mild low-density, progressively inhomogeneous enhancement	NA	Positive	Splenectomy	(19)
22	64	No clinical symptoms	60	NA	Well-defined, hypodense, with central necrosis and no signs of adjacent organ invasion	NA	Positive	Laparoscopic splenectomy	(20)

NA, unavailable data.

The sonographic features of splenic IPT-like FDCS may vary according to tumor size. For small lesions, the mass tend to appear as a well-circumscribed, uniformly hypoechoic nodule, with no detectable blood flow signals on color Doppler imaging. In contrast, larger lesions may exhibit heterogeneous echogenicity, accompanied by either well-defined or ill-defined margins, and intralesional blood flow signals can often be identified (10). Research focusing on the contrast-enhanced ultrasound (CEUS) manifestations of IPT-like FDCS remains relatively limited to date. In a study conducted by Xu et al., the lesion was noted to exhibit mild, rapid, heterogeneous enhancement that progressed centripetally from the periphery to the center during CEUS examination (21). For differential diagnosis, splenic hemangiomas typically present as well-circumscribed hypoechoic or hyperechoic lesions on conventional ultrasound, and their CEUS pattern is characterized by peripheral nodular enhancement—a feature that necessitates the inclusion of splenic hemangioma in the differential diagnosis of IPT-like FDCS (22).

Splenic IPT-like FDCS manifests as a hypodense lesion with either well-demarcated or indistinct borders on CT (23). Notably, the contrast enhancement patterns of IPT-like FDCS in the liver and spleen differ significantly. Hepatic tumors exhibit mild enhancement during the arterial phase, followed by gradual washout of contrast in the portal venous and delayed phases, ultimately demonstrating a density slightly lower than that of the adjacent hepatic parenchyma. In contrast, the solid components of splenic tumors display mild, persistent enhancement, while central regions with liquefactive necrosis remain non-enhancing (21).

Hepatic IPT-like FDCS may typically demonstrate hypointensity on T1-weighted image and hyperintensity on T2-weighted image. In contrast, splenic tumors more frequently exhibit isointense or hyperintensity on T1-weighted image and mild hypointensity on T2-weighted image, with heterogeneous internal signal intensity owing to fibrous scarring and vascular components. The solid portions display mild progressive enhancement, while central liquefactive or necrotic areas demonstrate no significant enhancement (11, 12). However, in our case, the tumor demonstrated a mildly hyperintense signal on T2-weighted image. This variation in T2-weighted image may be associated with the degree and distribution of inflammatory cells and proliferating capillaries within the tumor (13). In addition, a peripheral hypointense ring was visualized on T2-weighted imaging; this ring exhibited mild enhancement during the delayed phase of contrast-enhanced T1-weighted imaging. This radiological feature could potentially correlate with the vascular distribution within the tumor’s pseudocapsule.

Splenic IPT-like FDCS requires differentiation from inflammatory pseudotumor, hemangioma, and splenic lymphoma on MRI scans. Inflammatory pseudotumor of the spleen may present with slightly hypointense signals on T1-weighted image and slightly hyperintense signals on T2-weighted image, along with peripheral rim-like enhancement at arterial phase (24). Splenic lymphoma is often associated with splenomegaly and enlarged retroperitoneal lymph nodes, typically demonstrating homogeneous enhancement of the tumor parenchyma (25). Splenic hemangioma exhibits markedly hyperintense signals on T2-weighted image and shows gradual centripetal enhancement during contrast-enhanced phases (2). These differential diagnostic features are summarized in **Table 2** for clearer comparison.

**Table 2:** Comparison of imaging and clinical characteristics of splenic IPT-like FDCS and other similar diseases.

Feature	IPT-like FDCS	Inflammatory pseudotumor	Splenic hemangioma	Splenic lymphoma
T1WI signal	Isointense or hyperintensity	Slightly hypointense	Iso- to slightly hypointense	Iso- to slightly hypointense
T2WI signal	Mild hypointensity, with a hypointense curvilinear border surrounding the tumor	Slightly hyperintense	Markedly hyperintense	Mild to moderately hyperintense; homogeneous in most cases
Enhancement pattern	Solid portions display mild progressive enhancement	Peripheral rim-like enhancement in arterial phase; mild internal enhancement in delayed phase	Gradual centripetal enhancement from periphery to center in contrast-enhanced phases	Homogeneous enhancement of tumor parenchyma; no obvious rim enhancement
Typical clinical clues	Rare; may be asymptomatic or present with mild abdominal discomfort; no specific systemic symptoms	May be associated with local inflammation or infection; mild fever or elevated inflammatory markers	Benign lesion; usually asymptomatic; detected incidentally	Often accompanied by splenomegaly and retroperitoneal lymphadenopathy; may have B symptoms (fever, night sweats, and weight loss)

Histologically, IPT-like FDCS shares overlapping features with inflammatory pseudotumor. Microscopically, the neoplastic cells exhibit spindle-to-oval morphology and are organized in a fascicular, whorl, or storiform pattern (26). A defining histopathological distinction from classical FDCS is the prominent mixed inflammatory infiltrate (lymphocytes, plasma cells, and histiocytes) permeating the tumor microenvironment in IPT-like FDCS. Notably, the occasional presence of binucleated or multinucleated Reed–Sternberg-like cells may create diagnostic ambiguity, necessitating differentiation from Hodgkin lymphoma (9).

Immunohistochemically, CD21, CD23, and CD35 are regarded as key markers for confirming the follicular dendritic cell lineage, a view widely recognized by most scholars (27). In contrast, inflammatory pseudotumors lack consistent expression of FDC lineage markers and are often positive for myofibroblastic markers, while classical Hodgkin lymphoma is characterized by the expression of CD30 and CD15—these immunophenotypic differences constitute the key to differential diagnosis between these entities.

Compared with FDCS, IPT-like FDCS exhibits lower invasiveness and more favorable clinical outcomes (28). This subtype typically follows an indolent disease course, and complete surgical resection remains the standard therapeutic strategy for localized lesions. Current evidence on adjuvant radiotherapy and chemotherapy remains inconclusive, as their efficacy in the management of IPT-like FDCS remains controversial (29, 30). Furthermore, emerging data suggest limited responsiveness to contemporary immunotherapeutic regimens in this tumor subtype (31). Notably, longitudinal studies reveal post-treatment recurrence and metastasis rates of 28% and 27%, respectively, highlighting the necessity of rigorous long-term surveillance (32).

## Conclusion

Overall, IPT-like FDCS is an extremely rare malignant neoplasm that typically presents with nonspecific clinical manifestations. The present case provides novel insights into the multimodal imaging characteristics of this uncommon malignancy. Notably, the integration of multimodal imaging findings with pathological results is key to the accurate diagnosis of this rare neoplasm—imaging serves as a critical preoperative clue, while pathology confirms the definitive diagnosis. The radiological features of IPT-like FDCS are heterogeneous, with progressive enhancement on both CT and MRI serving as a helpful diagnostic clue for this entity. In addition, a peripheral hypointense ring on T2-weighted imaging may serve as a valuable imaging marker for the preoperative identification of IPT-like FDCS. Furthermore, EBV positivity, combined with the specific immunohistochemical profile (CD21/CD23/CD35 positivity), plays a crucial role in the differential diagnosis and definitive confirmation of the disease.

## Data Availability

The original contributions presented in the study are included in the article/**Supplementary Material**. Further inquiries can be directed to the corresponding author.
